# PANoptosis: A New Insight Into Oral Infectious Diseases

**DOI:** 10.3389/fimmu.2021.789610

**Published:** 2021-12-14

**Authors:** Weiyi Jiang, Zilong Deng, Xingzhu Dai, Wanghong Zhao

**Affiliations:** Department of Stomatology, Nanfang Hospital, Southern Medical University, Guangzhou, China

**Keywords:** PANoptosis, apoptosis, necroptosis, pyroptosis, oral infectious diseases, oral microbiomes

## Abstract

The oral microbiome, one of the most complex and intensive microbial ecosystems in the human body, comprises bacteria, archaea, fungi, protozoa, and viruses. Dysbiosis of the oral microbiome is the initiating factor that leads to oral infectious diseases. Infection is a sophisticated biological process involving interplay between the pathogen and the host, which often leads to activation of programmed cell death. Studies suggest that pyroptosis, apoptosis, and necroptosis are involved in multiple oral infectious diseases. Further understanding of crosstalk between cell death pathways has led to pyroptosis, apoptosis, and necroptosis being integrated into a single term: PANoptosis. PANoptosis is a multifaceted agent of the immune response that has important pathophysiological relevance to infectious diseases, autoimmunity, and cancer. As such, it plays an important role in innate immune cells that detect and eliminate intracellular pathogens. In addition to the classical model of influenza virus-infected and *Yersinia*-infected macrophages, other studies have expanded the scope of PANoptosis to include other microorganisms, as well as potential roles in cell types other than macrophages. In this review, we will summarize the pathophysiological mechanisms underlying inflammation and tissue destruction caused by oral pathogens. We present an overview of different pathogens that may induce activation of PANoptosis, along with the functional consequences of PANoptosis in the context of oral infectious diseases. To advance our understanding of immunology, we also explore the strategies used by microbes that enable immune evasion and replication within host cells. Improved understanding of the interplay between the host and pathogen through PANoptosis will direct development of therapeutic strategies that target oral infectious diseases.

## 1 Introduction

The oral microbiome is the general term used to describe the bacteria, fungi, and viruses that colonize the oral cavity. It includes both pathogenic bacteria/microbes and probiotics. Homeostasis of the oral microbiome plays a crucial role in maintaining health. Once the balance of the oral microbiota is upset, predominant pathogens can cause a variety of oral infectious diseases, including periodontitis, caries, pulp and periapical infections, and oral mucositis (1). According to The Systematic Analysis of Global Burden of Oral Conditions, oral infectious diseases are one of the most prevalent infectious diseases globally, leading to a heavy burden on both health care and economy (2). With the enormous progress in understanding the pathogenesis of oral infectious diseases, cell death is considered to be the vital mechanism for host defense and inflammation-mediated immune response. Therefore, more comprehensive understanding of pathogen-induced cell death provides underlying therapeutic targets for blocking uncontrolled inflammation, and deliver new insights into the prevention and treatment for oral infectious diseases in clinical setting.

Programmed cell death (PCD) is triggered when the immune system senses cellular stressors and microbial pathogens. Its role is to eliminate intracellular pathogens and maintain homeostasis. Pyroptosis, apoptosis, and necroptosis are three key PCD pathways characterized by certain molecular and genetic features (3). During pyroptosis, caspase-1 proteolytically activates Gasdermin-D (GSDMD) to release its N-terminal pore-forming domain from the auto-inhibitory C-terminal domain, which in turn results in pore formation in the plasma membrane. The GSDMD pore then mediates pyroptotic cell lysis, followed by release of intracellular cytokines (e.g., IL-1β and IL-18) and damage-associated molecular patterns (DAMPs) (4). Similarly, necroptosis occurs following receptor-interacting protein kinase (RIPK) 3-dependent phosphorylation of mixed lineage kinase domain-like protein (MLKL), which triggers translocation of MLKL to the plasma membrane and disrupts membrane integrity (5). Different from the two lytic cell death pathways, apoptosis is characterized by cell shrinkage and formation of apoptotic bodies, making it a non-inflammatory type of cell death. The apoptotic bodies are scavenged by phagocytes, thereby limiting the release of intracellular cytokines and inflammatory DAMPs. Apoptosis is instigated by cleavage of initiator caspases (caspase-8/9), followed by cleavage of executioner caspases (caspases-3/6/7) that, through cleavage of a broad range of substrate proteins, drive apoptotic cell death (6).

PANoptosis is a newly emerging concept that highlights crosstalk and co-ordination between these three pathways. This concept was first proposed by Malireddi in 2019 (7). Early studies of cell death focused on the unique programs and biochemical functions underlying each of these individual mechanisms; however, recent studies highlight crosstalk and redundancies among them. In cells with certain infection condition, such as influenza virus (IAV)-infected macrophages, the three types of cell death are not activated independently of each other; instead, they act simultaneously (8). Mutual regulation exists, and compensatory reactions will take place when one pathway is blocked. PANoptosis embodies cell death and inflammation, which are associated with homeostasis and autoinflammation upon microbial infection. It is universally acknowledged that microbial infection and the host immune response are involved in induction of oral infectious diseases. Accumulating preclinical and clinical evidence shows that pyroptosis, apoptosis, and necroptosis play roles in multiple oral infectious diseases (9–11). Additionally, animal experiments suggest that interventions targeting PANoptosis-related molecules have a significant impact on initiation and progression of oral infectious diseases (12–14). Infection by oral pathogens activates caspases and other effector molecules, resulting in cell death and cytokines release. Emerging and ongoing efforts have revealed many links between PANoptosis and oral infectious diseases, and integrating and summarizing these evidence can advance our understanding of the role of PANoptosis in oral infectious diseases, therefore helping to uncover the complex network which existed among multiple molecular events of the host’s response to those pathogens.

In this review, we focus on the mechanisms underlying activation and maintenance of PANoptosis in response to infection by oral pathogens, and the functional consequences. We discuss the possibility of manipulating the immune response as a therapeutic strategy, as well as the search for potential molecular targets for treatment and prevention.

## 2 Composition and Function of the PANoptosome

Increasing understanding of pathways involved in PCD has revealed co-regulation and crosstalk between pathways (15–18). Master regulators, a class of upstream molecules, regulate their downstream cascades simultaneously in these complex regulatory networks. For instance, Z-DNA binding protein 1 (ZBP1) and transforming growth factor beta-activated kinase 1 (TAK1) have essential roles in orchestrating multiple cell death pathways (7, 19). Activation of ZBP1 by pathogens (e.g., IAV infection) or inhibition of TAK1 induces pyroptosis, apoptosis, and necroptosis contemporaneously. How do ZBP1 and TAK1 regulate three cell death pathways at the same time? Based on the knowledge of cell death complex, such as the inflammasome and the necroptosome, researchers hypothesize that a multi-protein complex is assembled to form a molecular scaffold for contemporaneous engagement of key molecules from pyroptosis, apoptosis, and necroptosis. In agreement with above hypothesis, accumulating evidence from immunoprecipitation revealed widespread interaction and binding among ZBP1, RIPK3, RIPK1, caspase-8, ACS and Fas-associated protein with death domains (FADD). However, no clear mechanism for the domain-domain interaction has been deciphered. Phylogenetic analysis of the assembled domains gives us a hint as to how a multimeric complex can be organized (20). It was found that RIPK1 and RIPK3 interact with ZBP1 through their RIP homotypic interaction motif (RHIM), and then recruit caspase-8 *via* FADD; this occurs due to their high sequence similarity between the assembled domains (21, 22). The complex was subsequently termed as the PANoptosome (8). The specific components of the PANoptosome differ according to the stimulus. For example, during IAV infection, ZBP1 senses IAV and promotes interaction between RIPK3, RIPK1, caspase-8, caspase-6 and the NLRP3 inflammasome to form the ZBP1 PANoptosome complex; this complex drives PANoptosis (23–25). Herpes Simplex virus 1 (HSV-1) or *Francisella novicida* is sensed by the AIM2 PANoptosome which then regulates the immune response. In this case, unlike above ZBP1 PANoptosome, AIM2, pyrin and ZBP1 are members of the PANoptosome, along with ASC, caspase-1, caspase-8, RIPK3, RIPK1 and FADD (26).

In general, compositions of the PANoptosome can be grouped into three different classes based on their biological and chemical functions: including sensors (e.g., ZBP1 and NLRP3), adaptors (e.g., ASC and FADD) and catalytic effectors (e.g., RIPK1, RIPK3, caspase-1 and caspase-8) (20). In this review, we will focus on sensors and catalytic effectors, which serves as major contributors in PANoptosome.

### 2.1 Sensors

Structurally, ZBP1 possesses two Z-nucleic acid binding domains (Zα1 and Zα2) at its N-terminus, two RIP homotypic interaction motifs (RHIM1 and RHIM2) in the middle of the protein sequence, and a conserved C-terminal domain. Not only do the Z-DNA binding domains bind Z-DNA, B-DNA, and RNA, they are also considered to sense endogenous nucleic acid ligands (27–29). The RHIM1 domain of ZBP1 mediates its interactions with other RHIM domain-containing proteins (RIPK1 and RIPK3). The conserved C-terminal domains of ZBP1 interact with TBK1 and IRF3 to induce type I IFN responses to immunostimulatory DNA (19, 30). Therefore, ZBP1 is characterized as a critical innate immune sensor and as a central regulator of cell death and inflammatory responses. For example, during influenza A virus (IAV) infection, ZBP1 senses the virus, initiates assembly of the PANoptosome, and triggers PANoptosis. ZBP1 recruits RIPK3 and caspase-8 to activate the ZBP1-NLRP3 inflammasome, thereby inducing pyroptosis and secretion of IL-1β and IL-18 (23). In parallel, the ZBP1-RIPK3 complex activates caspase-8-dependent apoptosis and MLKL-mediated necroptosis (31). The Zα2 domain of ZBP1 plays a role in sensing; indeed, mutation of the Zα2 domain blocks virus recognition, and also blocks activation of NLRP3 inflammasome (32). Collectively, ZBP1 is an innate immune sensor that recognizes IAV, initiates assembly of the PANoptosome, and triggers PANoptosis and inflammation.

In addition, NLRP3, another known sensor, has been increasingly and deeply investigated in the past two decades. As a member of NOD-like receptor (NLR) family, the NLRP3 inflammasome has been reported to be activated by several stimuli, including endogenous danger signals, ionophores like nigericin, particulate matters such as silica and uric acid crystals, viral infection, Gram-positive bacterial infection, as well as Gram-negative bacterial and fungal infection such as Citrobacter rodentium, Aspergillus fumigatus. However, there are no sufficient data supporting that NLRP3 directly binds to these diverse ligands (33). Additionally, whether NLRP3 plays the role as a sensor to trigger PANoptosis still remains unknown, although it has been classified as an underlying sensor during PANoptosis, as proposed by Kanneganti et al. (20).

### 2.2 Effectors

RIPK1 is classified as a catalytic effector because it has an amino-terminal kinase domain. Earlier studies show that RIPK1 kinase activity is needed for RIPK3/MLKL-mediated necroptosis, and subsequent inflammatory responses during an immune response (34, 35). In addition, RIPK1 has RHIM and death domain (DD) at its carboxy-terminus, indicating the potential scaffold function of RIPK1. The RHIM and DD mediate interactions with other RHIM- or DD containing proteins, such as RIPK3, TRIF, FADD, and ZBP1. Several studies have addressed the occurrence of RIPK1 kinase activity-independent necroptosis in TAK1-deficient macrophages (21, 36). TLR priming in TAK1 deficiency cells induces necroptosis bypassing the requirement for RIPK1 kinase activity (21). These data indicate that RIPK1 acts as an adaptor molecule (37). In addition to its role in speeding up cell death, the scaffold function of RIPK1 promotes cell survival signals. Its protective role in homeostasis was proved in conditional gene knockout mice. Intestinal epithelial cell (IEC)-specific knockout of RIPK1 causes IEC apoptosis and RIPK3-dependent IEC necroptosis. Similar functions of RIPK1 have also been shown to present in epidermis-specific RIPK1 knockout mice, which resulted in severe skin inflammation with apoptosis and necroptosis of keratinocytes. (38). These findings consistently demonstrated that regulation of PANoptosis by RIPK1 is essential for homeostasis, cell death, and inflammation through kinase-dependent and -independent functions.

Caspase-8 mediates crosstalk between apoptosis, pyroptosis, and necroptosis (39). It plays multiple roles in cell death by 1) mediating receptor-mediated apoptosis (40, 41); 2) regulating canonical and non-canonical inflammasome activation during pyroptosis (42, 43); and 3) inhibiting activation of RIPK3-driven necroptosis (44). Early in 1998, Stennicke et al. showed that caspase-8 activates the death-inducing signaling complex and then cleaves pro-caspase-3, leading to extrinsic apoptosis in response to FAS-activation (45). Studies demonstrate that caspase-8 is crucial for canonical and non-canonical inflammasome activation. Caspase-8 and its adapter FADD are required for both priming and activation of the NLRP3 inflammasome in macrophages (42). In addition, caspase-8 plays a direct role activating the RIPK1/FADD/caspase-8 complex, and drives GSDMD cleavage and pyroptosis, independent of caspase-1 (46). In addition, loss of caspase activity of caspase-8 leads to activation of necroptosis, which results in large amounts of cell death, inflammation, and embryonic lethality through activation of RIPK1 and RIPK3/MLKL (44). Caspase-8-mediated cleavage of RIPK1 is a critical event that limits RIPK1-dependent cell death and inflammation (47). Collectively, the data suggest that caspase-8 is a central component of the PANoptosome, and that it plays a role in different forms of cell death during innate immune responses.

Caspase-6 acts as an effector caspases during apoptosis (48). In addition, caspase-6 promotes activation of PCD pathways and plays an essential role in host defense against IAV infection. Loss of caspase-6 from bone marrow-derived macrophages (BMDMs) leads to reduced ZBP1-mediated PCD pathways upon IAV infection. However, it is unclear whether caspase-6 influences PANoptosome assembly and it has remained a mystery for decades. A recent study identified caspase-6 as a critical component of the PANoptosome in that it promotes assembly of the PANoptosome by reinforcing the interaction between RIPK3 and ZBP1 within the complex, suggesting a novel scaffold function for this presumed apoptotic executioner caspase. Caspase-6 facilitates the RHIM-dependent binding of RIPK3 to ZBP1 *via* its interaction with RIPK3 (25).

### 2.3 Regulators

Although TAK1 is not a component of the PANoptosome, it acts as a master regulator of PANoptosis. Researchers discovered that TAK1 plays a critical role in the cell death process by promoting cell survival *via* its negative effect on activation of PANoptosis. Under physiological conditions, inhibitory phosphorylation of RIPK1 by TAK1 is critical for blocking spontaneous activation of PANoptosis (49). BMDMs lacking TAK1 undergo spontaneous cell death; TAK1-deficient cells form a complex consisting of RIPK1 and caspase-8 through DD interactions, thereby triggering RIPK1-dependent PANoptosis. This complex promotes FADD/caspase-8-dependent apoptosis through activation of effector caspase-3 and -7, and promotes necroptosis *via* RIPK3-mediated phosphorylation of MLKL (7). A study demonstrated that the absence of TAK1 induces spontaneous RIPK1-dependent NLRP3 inflammasome activation and pyroptosis (50). Under priming conditions, mice harboring specific deletion of TAK1 in myeloid cells are significantly more susceptible to LPS-induced septic shock, with higher amounts of IL-1β, IL-6, and TNF-α in plasma than wild-type mice (51). During infection, the intracellular bacterium *Yersinia* produces the toxin YopJ to inactivate TAK1 (52, 53). Pathogen-mediated inhibited of TAK1 activates the RIPK1–FADD–caspase-8 complex, which further leads to caspase-8-dependent cleavage of GSDMD and cell death (54). These data demonstrate that TAK1 plays an essential regulatory role in inhibiting cell death pathways, maintaining cellular homeostasis and defending against microbes during innate immune responses.

## 3 Potential Role for PANoptosis During Oral Infectious Diseases

**Figure 1:**
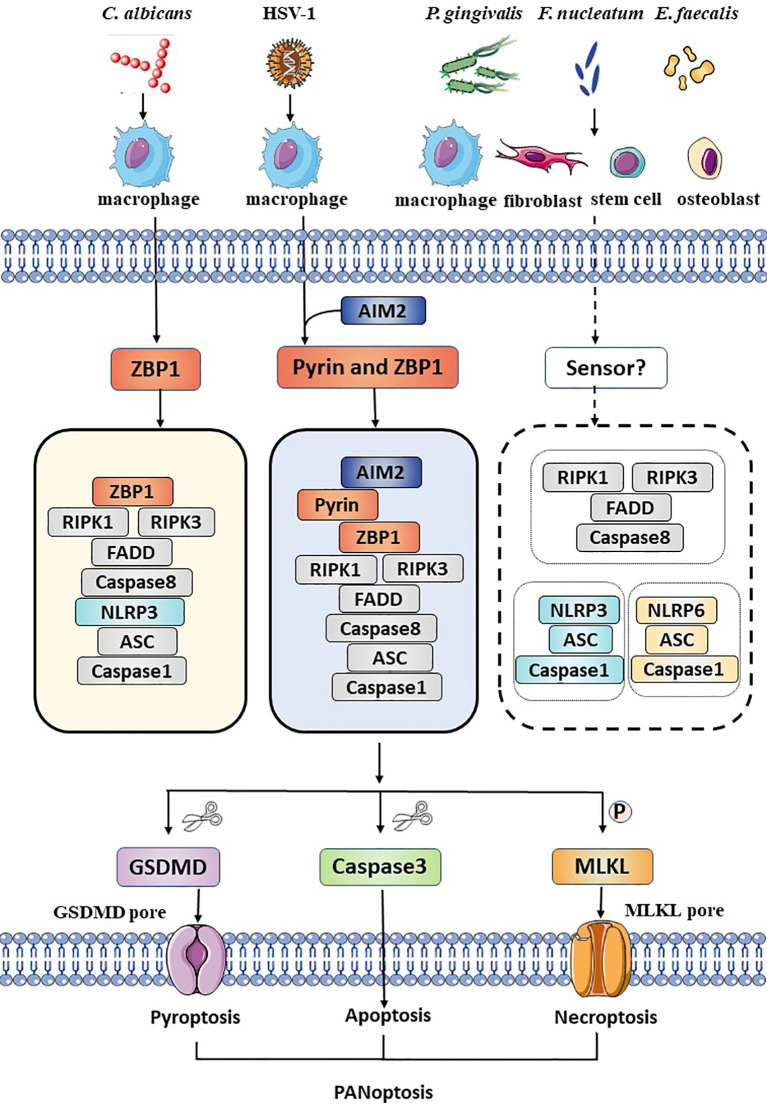
Schematic summary of the activation of PANoptosis in several kinds of cells during oral pathogens infection. During *Candida albicans* infection, ZBP1 senses the pathogen and recruits RIPK3, RIPK1, and caspase-8 to assemble the ZBP1 PANoptosome, causing GSDMD-mediated pyroptosis, caspase-8-dependent apoptosis and MLKL-mediated necroptosis (PANoptosis). DNA virus, HSV-1, is sensed by ZBP1 and pyrin and actives the AIM2 PANoptosome which inducing PANoptosis in macrophages. Bacteria such as *Porphyromonas gingivalis*, *Fusobacterium nucleatum*, and *Enterococcus faecalis* active pyroptosis, apoptosis, necroptosis and release intracellular cytokines and DAMPs not only in immune cells, but also in fibroblasts, stem cells and osteoblasts. However, the exact sensor is unclear. The NLRP3 inflammasome, the NLRP6 inflammasome and the necroptosome are involved, but the interaction among these molecules remains unknown.

Oral infectious diseases include dental caries, periapical periodontitis, periodontal disease, oral mucosal disease, and other soft tissue and space infections of the cranio-maxillofacial region. Herein, we summarized the details for the pathogen-induced PANoptosis in different oral infectious diseases, as well as related pathogens, involved modulators and pathways (**Figure 1** and **Table 1**).

**Table 1:** Summary of cell death pathways modulated by pathogens in oral infectious diseases.

Oral disease	Pathogen	Specific ligand	Function	Cell death pathway	Cell/tissue type	Reference
Oral mucosal disease	*Candida albicans*	unknown	promote	ZBP1-RIPK3/NLRP3/caspase-8-mediated PANoptosis	mouse bone marrow derived macrophages (BMDMs)	(32)
		unknown	promote	Dectin-1-RIPK1/RIPK3/MLKL-mediated necroptosis	mouse peritoneal macrophages	(55)
	Herpes simplex virus 1	ICP0 Gene Expression “apoptoxin”	promote	caspase-3-mediated apoptosis	human epithelial cells (HEp-2)	(56)
		ribonucleotide reductase ICP6	promote	RIPK3/MLKL-mediated necroptosis	mouse fibroblasts (L929)	(57)
		RNA transcripts	promote	ZBP1-mediated necroptosis	mouse lymphatic endothelial cells (SVEC4-10)	(58)
		unknown	promote	AIM2-, ZBP1- and pyrin-mediated PANoptosis	BMDMs; human THP-1 macrophages	(26)
		ribonucleotide reductase ICP6	inhibit	RIPK3/MLKL pathway	human colon adenocarcinoma cells (HT-29)	(59)
		HSV-1 tegument protein VP22	inhibit	AIM2/caspase-1 pathway	mouse macrophages (J774A.1), human THP-1 macrophages	(60)
Periodontal disease	*Porphyromonas gingivalis*	unknown	promote	non-caspase-dependent apoptosis	human gingival fibroblasts (GFs)	(61)
		LPS	promote	NLRP3/caspase-1/GSDMD-mediated pyroptosis	GFs	(62)
		OMV	promote	mitochondrial disfunction and NLRP3/caspase-1-mediated pyroptosis	BMDMs; human monocyte-derived macrophages	(63)
		unknown	promote	NLRP6/caspase-1/GSDMD-mediated pyroptosis	GFs	(64)
		unknown	promote	RIPK1/RIPK3/MLKL-mediated necroptosis	human periodontal ligament fibroblasts	(10)
		HmuY	promote	caspase-7-related apoptosis	peripheral blood mononuclear cells (PBMCs)	(65)
		unknown	promote	lysosomal destruction-NLRP3 and AIM2/caspase-1 pathway	human THP-1 macrophages	(66)
		unknown	promote	NLRP3/Caspase-4 and NLRP3/Caspase-1	human THP-1 macrophages	(11)
		unknown	promote	RIPK1/RIPK3/MLKL-mediated necroptosis	human THP-1 macrophages	(67)
		unknown	promote	NLRP3 inflammasome-mediated pathway	human gingival mesenchymal stem cells	(68)
		LPS	promote	NLRP3 inflammasome and RIPK1/RIPK3/MLKL-mediated necroptosis	human periodontal ligament stem cells	(69)
		NDKs	inhibit	P2X7-mediated apoptosis	human gingival epithelial cells	(70)
		NDKs	inhibit	caspase-1-mediated pyroptosis	human gingival epithelial cells	(71)
		gingipains: RgpA, RgpB, and Kgp	inhibit	caspase-1-mediated pyroptosis	human THP-1 macrophages	(72)
		fimbriae	inhibit	P2X7 receptor-eATP-mediated inflammatory responses	BMDMs	(73)
Pulp and periapical diseases	*Fusobacterium nucleatum*	heat-labile surface protein	promote	caspases-mediated apoptosis	PBMCs; human polymorphonuclear cells; human U937 monocytes; human THP-1 macrophages	(74)
		unknown	promote	NLRP3/caspase-1-mediated pathway	human THP-1 macrophages	(75)
		unknown	promote	RIPK3/MLKL-mediated necroptosis	periapical tissue of Balb/c mice; mouse fibroblasts (L929)	(14)
		OMV	promote	FADD/RIPK1/RIPK3/caspase-3-mediated necroptosis	PBMC-derived macrophages and human colonic epithelial cells	(76)
	*Enterococcus faecalis*	unknown	promote	Bax/Bcl-2/caspase-3-mediated apoptosis	human osteoblastic cells (MG63 cells)	(77)
		LTA	promote	NLRP3/caspase-1-mediated pathway	mouse macrophages RAW264.7	(78)
		unknown	promote	NLRP3/caspase-1-mediated pyroptosis	periapical tissue of Sprague-Dawley rats; MG63 cells	(77)
		unknown	promote	RIPK3/MLKL-mediated necroptosis	MG63 cells	(79)

### 3.1 Oral Mucosal Disease

The oral mucosa is a primary barrier site that is in direct contact with microbes. Mucosal health is based on a homeostatic balance between the tolerance of the host and colonization by microbiota. Oral mucosal disease is one of the most common oral diseases observed in the clinic. *Candida albicans* (*C. albicans*) is the most common opportunistic pathogen in the oral cavity, although it exists in many parts of the human body, including the upper respiratory tract, vagina, and intestinal tract, even in healthy people. However, when the balance of the normal flora is upset, or an individual is immunocompromised, overgrowth of mucosal *C. albicans* can occur, resulting in development of oropharyngeal candidiasis (also known as thrush). The infection rate is higher in infants, the elderly, antibiotic abusers, and cancer patients receiving chemoradiation therapy (80, 81). HSV-1 is a DNA virus and a common human pathogen that infects around 80% of adults. The virus not only causes acute infectious disease of the oral mucosa and peroral skin, but also establishes a latent infection in sensory neurons that lasts for the entire life of the host (82).

#### 3.1.1 *C. albicans* Induces PANoptosis in Murine Macrophages

Researchers have shown that *C. albicans* infection induces PCD in the form of PANoptosis, with releasing high levels of cytokines. Previous evidence suggests that fungal DNA, spores, and cell wall-associated polysaccharides are recognized by inflammasome sensors such as NLRP3 and NLRC4. Activation of these inflammasome is important for control of mucosal Candida infection and impacts inflammatory cell recruitment to infected tissues, as well as protects against systemic dissemination of infection. (83). In addition to bacterial flagellin and T3SS proteins, mitochondrial DNA is thought to activate NLRC4 during *Pseudomonas aeruginosa* infection (84). Collectively, it is plausible that damage-induced release of mitochondrial DNA might activate the NLRC4 inflammasome to drive immune response under certain pathogen challenges (83, 84). Additionally, *C. albicans* can induce necroptosis in macrophages; studies show that Dectin-1 is a sensor for fungal PAMPs in myeloid cells, and that it subsequently induces necroptosis through the RIPK1/RIPK3/MLKL cascade (55). Further studies have focused on the components of PANoptosis. ZBP1 was identified as an initial sensor that activates PANoptosis. Markers of PANoptosis are reduced in Zbp1^–/–^ and Zbp1^ΔZα2/ΔZα2^ BMDMs, which indicates that the Za2 domain of ZBP1 plays crucial roles in sensing and driving downstream signaling pathways. Deletion of ZBP1 abolishes NLRP3 inflammasome activation completely and reduces the release of cytokines. Interestingly, similar results were observed for Casp1/11^–/–^Ripk3^–/–^Casp8^–/–^ BMDMs. It is worth mentioning that the intrinsic relationship between these three death pathways was revealed preliminarily. Loss of pyroptotic proteins (caspase-1 and GSDMD) has minimal effect on apoptosis, whereas activation of necroptosis is increased in response to *C. albicans* infection. Deficiency of MLKL or RIPK3 has a minor impact on pyroptosis and apoptosis. Caspase-8 not only regulates NLRP3 inflammasome activation, but also negatively regulates the necroptotic pathway (85). Taken together, these findings suggest that PANoptosis is crucial for mediating inflammatory cell death and cytokines release during fungal infection. Combined deletion of RIPK3, caspase-1, and caspase-8, which blocks all three arms of PANoptosis, rescues cells from death, whereas inhibition of a single arm does not.

PANoptosis functions in response to *C. albicans* infection, but the underlying molecular mechanism is controversial. There are four questions remain unsettled: 1) Which molecule is the inducer: fungal PAMPs (fungal DNA, spores, cell wall-associated polysaccharides), host-derived DAMPs (ROS and ATP), or host-synthesized endogenous RNA and DNA? 2) Which is the sensor: ZBP1, the co-regulator of three arms of PANoptosis, the inflammasome, or Dectin-1? 3) Although deletion of ZBP1 protects cells from PCD, can it also prevent mitochondrial damage caused by fungal infection? and 4) In addition to NLRP3, can ZBP1 regulate activation of NLRC4, AIM2, and other inflammasomes?

#### 3.1.2 Herpes Simplex Virus 1 Induces PANoptosis in Macrophages

Macrophages are important cells in innate immune, having many functions such as presenting antigen, eliminating intracellular pathogens and secreting various cytokines. Recently, efforts have been made to uncover the role and molecular mechanism of PANoptosis in macrophages (BMDMs and THP-1) during HSV-1 infection (26). It was found that HSV-1 infection induces PANoptosis in a manner dependent on AIM2 and the coordinated activation of pyrin and ZBP1. Mechanistically, AIM2 regulates the expression of pyrin and ZBP1 and forms a complex along with ASC, caspase-1, caspase-8, RIPK3, RIPK1 and FADD, namely, the AIM2 PANoptosome. AIM2 PANoptosome is required for activation of downstream effectors, inducing PANoptosis and cytokines release. In summary, HSV-1 can induce AIM2-, ZBP1- and pyrin-mediated PANoptosis in murine and human macrophages. This study revealed the activation of PCD in macrophages in host defense against HSV-1. Such presence of multiple stimulation-dependent PANoptosomes indicates the complexity and heterogeneity for the responses and regulations of innate immunity under various pathogen challenges and pathological conditions.

With the accumulating efforts, it is becoming clear about the detailed cell death pathways and involved modulators during HSV infection. Hosts induce three types of pathways (pyroptosis, apoptosis, and necroptosis) to fulfill its defensive functions against HSV-1. Apoptosis and cell death cascades are triggered by HSV infection and the protein coded by HSV-1 α0 gene acts as an “apoptoxin” that is necessary to trigger apoptosis (56). Besides, HSV-1 triggers RIPK3/MLKL necroptosis *in vitro* and *in vivo*. Its viral protein ICP6 triggers necrosome assembly and drives activation of necroptosis in mouse cells (57). Mechanistically, an ICP6 dimer/oligomer recruits RIPK1 and RIPK3, or two RIPK3 molecules, to form RIPK1-RIPK3 hetero- or RIPK3-RIPK3 homo-dimers. Later, scholars found that HSV-1 infection triggers necroptosis through ZBP1. ZBP1 functions as a sensor that detects nascent RNA transcripts in murine macrophages (58).

Intriguingly, although PANoptosis is the hosts’ strategy to control over intracellular pathogens and eliminate infected cells, it is also the targets regulated by pathogens for its immune escape. It is reported that HSV-1 regulates cell death during infection by suppressing necroptosis (86) as well as apoptosis (87). The mechanism of cell death suppression is mediated by the viral protein ICP6, which blocks caspase-8-mediated apoptosis as well as RIPK3-mediated necroptosis (86, 88). As mentioned above, ICP6 mediates the bonding between RIPK1 and RIPK3. In HSV-1 infected human cells, this interaction prevents the formation of necrosome and downstream necroptosis. However, in mouse cells, such interactions of ICP6 are proved to have an ability to trigger necroptosis (59). Since HSV-1 is a natural human pathogen, it is possible that the RHIM of HSV-1 has already evolved to evade RIPK1 and RIPK3-mediated necroptosis in human cells, but not in mouse cells. Maruzuru et al. suggested that HSV-1 tegument protein VP22 inhibits AIM2-dependent inflammasome activation by interacting with AIM2 and interfering oligomerization of AIM2 (60). Collectively, HSV-1 has evolved various strategies to evade host antiviral innate immunity.

#### 3.1.3 PANoptosis Plays a Role in Host Defense in Oral Mucosal Disease

PANoptosis protects the oral cavity of mice from infection by *C. albicans*; mice lacking NLRP3 are hypersusceptible to *C. albicans* infection and carry substantially higher fungal burdens in the kidney than wildtype (WT) mice (89). Compared with WT controls, mice lacking NLRC4 have an elevated oral fungal burden and substantially increased susceptibility to dissemination of the fungus (83) These data indicate that inflammasome-mediated pyroptosis is important for control of mucosal *C. albicans* infection and impacts inflammatory cell recruitment to infected tissues, as well as protects against systemic dissemination of infection. In addition, induction of necroptosis signaling is critical for host defense against *C. albicans* infection *in vivo*, even in the presence of caspase-8 (55). In this regard, PANoptosis is important for initiation of an immune response to aid clearance of infection. With respect to viruses, PANoptosis confers host protection against infection. Viruses are obligatory intracellular pathogens, and their propagation is fully dependent on the intracellular milieu of the host cell. Therefore, they have developed elaborate countermeasures to avoid or delay host cell death. This is particularly true for large DNA viruses, which generally replicate with slower kinetics than RNA viruses. For example, ZBP1-mediated necroptosis plays a vital role in restricting HSV-1 propagation in mice. ZBP1 in HepG2 hepatocellular carcinoma cells can suppress viral replication through its Zβ and D3 domains. HSV-1 replication is enhanced by ZBP1 knockdown (90).

So far, most studies support that PANoptosis plays a protective role against C. albicans and HSV-1 infections of the oral mucosa. However, limitations exist in these studies, for the impact of PANoptosis was mainly based on innate immune cells (e.g. macrophages and dendritic cells). Since mucosal immunization is a complicated process appearing as a spectrum of tissue reactions, including degeneration of epithelial cells, infiltration of lymphocytes, dendritic cells and macrophages, different types of cells in mucosal tissue (e.g., oral epithelial cells, oral submucosal fibrosis cells and lymphocytes) should be taken into consideration when exploring the mechanisms of pathogenesis during mucosal infections. Encouragingly, a more recent report showed that TNF-α and IFN-γ can trigger PANoptosis in human cancer cell lines (91). These data suggest that PANoptosis can be activated not only in immune cells but also in non-immune cells. Therefore, it is worth investigating specific mechanisms of PANoptosis under certain stimulation in different cell types or pathologic conditions.

An emerging finding revealed that in addition to the direct role of infections, infection-mediated cytokines release could induce PANoptosis as well (92). Karki et al. found that TNF-α and IFN-γ released by innate immune cells activate the JAK/STAT1/IRF1 axis and induce nitric oxide production, thus driving caspase-8/FADD-mediated PANoptosis during SARS-CoV-2 infection. Above uncontrolled cell death can exacerbate tissue injury and compromise lung function. This study advances our understanding of PANoptosis, which could not only be activated by pathogen infection, but also be induced by some specific pro-inflammatory cytokines. It spurs us to ponder whether cytokine-induced PANoptosis occurs in oral mucosal infections, and whether it has a similar damaging influence on oral mucosa tissue. However, there are no further reports and literatures on the cytokine-induced PANoptosis in oral diseases at present. More related researches are awaited to address this gap in the future.

### 3.2 Periodontal Disease

Periodontal disease, which manifests as chronic inflammation of the periodontal support structure, includes gingivitis and periodontitis. The disease process is characterized by reversible inflammatory lesions in the soft tissues, followed by irreversible bone resorption and tooth loss. The initiating factor is dental plaque (a biofilm formed by specific bacteria that accumulate on the tooth surface and interact with host cells that release inflammatory mediators, evade host immune defenses, and are resistant to drugs).The “Red complex” comprises the most common pathogenic bacteria associated with periodontitis (*Porphyromonas gingivalis* (*P. gingivalis*), *Tannerella forsythia*, and *Treponema denticola*) (93). Among them, *P. gingivalis* is the major pathogen involved in the pathogenesis of periodontal disease (94).

After *P. gingivalis* infection, the first step of periodontal disease is bacterial invasion of gingival epithelial cells and destruction of the epithelial barrier. Then, recruited inflammatory cells release inflammatory mediators that degrade the matrix and kill tissue structural cells. Meanwhile, stem cells differentiate into multiple cell types to repair tissue damage. That is why studying cell death in fibroblasts, macrophages, and stem cells is important in the context of periodontal disease.

#### 3.2.1 *P. gingivalis* May Induce PANoptosis in Fibroblasts, Macrophages, and Stem Cells

Fibroblasts are the major cell type found in periodontal connective tissue; these cells comprise gingival fibroblasts (GFs) and periodontal ligament fibroblasts (PDLFs). Fibroblasts upregulate production of inflammatory mediators during development of periodontal disease. Desta and Graves found that *P. gingivalis* strain W83 infection induces apoptosis of GFs *via* non-caspase-dependent pathway (61). A study revealed simultaneous existence of pyroptosis. TEM analysis of *P. gingivalis*-infected GFs revealed lytic morphological features (cytoplasmic, nuclear, and mitochondrial swelling; membrane rupture; and chromatin condensation). Also, expression of mRNA encoding GSDMD, NLRP3, caspase-4, IL-1β, and IL-18 was upregulated after exposure to *P. gingivalis*-LPS (62). Interestingly, in addition to the classic NLRP3 inflammasome pathway, the NLRP6 inflammasome was also activated when GFs were infected by *P. gingivalis* strain W83. Pro-caspase-1 and pro-GSDMD were cleaved into their mature forms, which then formed pores in membranes and released pro-inflammatory mediators IL-1β and IL-18. Cell death was attenuated by NLRP6 knockdown or caspase-1 inhibition (64). Shi et al. claimed that necroptosis was observed only in PDLFs infected with live *P. gingivalis* at a high MOI. They showed that pMLKL, a marker of necroptosis, was observed at an MOI of 400 but not at lower MOIs. Surprisingly, silencing MLKL alone reduced cell death, whereas RIPK1 silencing increased cell mortality, and RIPK3 knockdown had no significant effect (10). This result indicates that RIPK1 and RIPK3 have other functions in regulating cell death. Although RIPK1 and RIPK3 are upstream signals of MLKL during necroptosis, they are critical components of the PANoptosome, which regulates multifaceted signaling platforms rather than a single defined pathway.

However, some scholars report different conclusions. For example, *P. gingivalis*-LPS did not induce necroptosis in GFs. There were no significant changes in expression of RIPK3 and MLKL mRNA after exposure to *P. gingivalis*-LPS (62). This study has some limitations, however. For example, RIPK3 and MLKL are mainly activated by phosphorylation, and the transcription level may not change significantly during necroptosis. Therefore, one cannot exclude necroptosis only at the mRNA level. The dose of *P. gingivalis* and the duration of challenge may be critical parameters that determine whether, and which type of, cell death is induced. As mentioned above, necroptosis was observed only after infection with a high MOI. Therefore, we suppose that *P. gingivalis* may induce fibroblast PANoptosis.

Macrophages are phagocytic innate immune cells that are important mediators of microbial infection. In response to tissue injury, macrophages become activated by specific signals from the damaged microenvironment. In addition, they are the primary cell type that is targeted by most species of intracellular bacteria. Apoptosis is the earliest and most thoroughly studied mode of PCD during *P. gingivalis* infection. A wealth of evidence supports the role of apoptosis during infection, and needs no further explanation here (95, 96). Some reports show that *P. gingivalis* strain 381 induces production of IL-1β by THP-1 cells through activation of caspase-1 and NLRP3 and AIM2, which is mediated by ATP release, P2X7 receptor ligation, and lysosomal destruction (66). A study discovered the link between the canonical and non-canonical pathways: activation of the NLRP3 inflammasome by caspase-4 or caspase-1 is dependent upon the type of periodontal microbe (11). Studies also report that activation of caspase-1 by *P. gingivalis* is triggered at an MOI of 10 and 100, but not 500 (72). In 2016, Ke et al. was the first to report that necroptosis of THP-1 cells was induced by *P. gingivalis* both *in vivo* and *in vitro*. Cells underwent necroptosis through activation of the RIPK1/RIPK3/MLKL signaling pathway. Pretreatment with inhibitors of RIPK1 and MLKL, or silencing of RIPK3 and MLKL, reduced the rate of cell death, accompanied by attenuated expression of TNF-α and IL-6 (67). Furthermore, Gram-negative bacteria can shed outer membrane vesicles (OMVs), which are thought to be a strategy to kill host cells and increase pathogenicity. *P. gingivalis* sorts bacterial DNA into OMVs, which are then delivered to bystander cells where they initiate mitochondrial disfunction *via* NLRP3-caspase-1-mediated pyroptosis and RIPK1/RIPK3-mediated necroptosis (63).

Stem cells play an essential role in tissue regeneration. Han et al. demonstrated that *P. gingivalis* inhibits mesenchymal stem cells (MSCs) by activating the NLRP3 inflammasome. *P. gingivalis* inhibits MSC migration, ALP activity, mineralization, and proliferation significantly, whereas NLRP3 inhibitors restore these functions (68). In addition to MSCs, PDLSCs are ideal candidate cells that drive periodontal tissue regeneration. Necroptosis in LPS-treated PDLSCs was detected by TEM investigation, immunofluorescence analysis, and flow cytometry analysis. It is worth noting that Nec-1 treatment not only inhibits the degree of necroptosis by inhibiting RIPK3 recruitment to RIPK1, but also ameliorates osteogenic differentiation with increased ectopic regeneration of cementum-like structures in nude mice (69).

#### 3.2.2 PANoptosis Is Associated With Immune Escape of *P. gingivalis*

Some microorganisms have evolved elaborate virulence factors to ensure their survival within the oral environment without eliciting an immediate and destructive host immune response. As a successful facultative intracellular organism, *P. gingivalis* has evolved an array of virulence factors and associated mechanisms that allow it to survive and spread in gingival tissue, and to evade immune surveillance. Prominent virulence factors include a secreted homologue of nucleoside-diphosphate kinases (NDKs), gingipains, and fimbriae.

NDKs derived from intracellular *P. gingivalis* are important for maximal inhibition of ATP-triggered P2X7-mediated apoptosis of infected gingival epithelial cells, as well as for ATP-induced generation of cellular ROS (70). Since ROS generation contributes to inflammasome activation, stimulation by ATP alone could activate pyroptosis in GECs; however, infection by *P. gingivalis* ameliorates the effects of ATP. Indeed, *ndk*-deficient *P. gingivalis* produces higher levels of activated caspase-1 and IL-1β. This suggests that the NDK homologue in *P. gingivalis* inhibits both caspase-1 activation and secretion of pro-inflammatory cytokines (71).

*P. gingivalis* secretes various peptidases, including three kinds of gingipains: RgpA, RgpB, and Kgp. Secretion of these proteases is mediated by the type IX secretion system (T9SS), a sophisticated protein secretion machinery. The three gingipains are thought to evade host immune responses *via* disruptive and evasive proteolytic actions. Interestingly, gingipains may also affect inflammasome activation. Recently, Jung et al. showed that the simultaneous protease activity of Kgp and RgpA and RgpB attenuates the caspase-1-activating potential of *P. gingivalis* in macrophages (72). Gingipains secreted from *P. gingivalis* degrade the released caspase-1 and IL-1β. Inflammasome activation upon infection with *P. gingivalis* is independent on gingipains; indeed, this is one of the evasion systems that enable bacteria to evade host immune responses.

Bacterial components such as fimbriae are capable of triggering inflammatory responses. Fimbriae activate NF-ĸB *via* TLR2 and induce production of pro-inflammatory cytokines such as TNF-α, IL-1β, IL-8, and IL-6. Researchers identified a role for fimbriae during anti-inflammatory responses (73). WT *P. gingivalis* and a fimbria-mutant form of *P. gingivalis* barely triggered secretion of mature IL-1β by macrophages in the absence of eATP. In the presence of eATP, challenge with non-fimbriated *P. gingivalis* elicited greater secretion of IL-1β than WT *P. gingivalis*. So, the authors concluded that the fimbriae of *P. gingivalis* inhibit ATP-induced secretion of IL-1β by macrophages through the P2X7 receptor.

The apparent paradoxical effects of bacteria (i.e., both triggering and suppressing) on host immune responses are thought to help them achieve persistent colonization in host tissues. Pathogens participate in and shape development of the host immune system, thereby reflecting the co-evolution of both. Host cells employ several ways (pyroptosis, apoptosis, and necroptosis) to fight bacteria. Pathogens, meanwhile, employ strategies to block activation of PANoptosis.

#### 3.2.3 PANoptosis Is A Potential Pathogenic Mechanism in Periodontal Disease

RNA sequencing of clinical samples revealed that one of the highest upregulated gene clusters in gingival tissues (97) and peripheral blood monocytes (98) from patients with chronic periodontitis (CP)is that related to apoptosis and cell death. Higher expression of NLRs (e.g., NLRP3, NLRP6, and NLRC4) and caspase-1 in gingival tissues from CP patients implies activation of pyroptosis (11). Notably, elevated levels of RIPK1, phosphorylated RIPK3, MLKL, phosphorylated MLKL, and FLIPL are common in gingival epithelia and connective tissues from CP patients, indicating the presence of necroptosis during progression of periodontitis (10). Importantly, increased expression of IL-1β in gingival crevicular fluid correlates significantly with the severity of periodontitis (13). Thus, PANoptosis is thought to be one of the pathogenic mechanisms underlying persistent inflammation observed in periodontal disease.

Experiments in a mouse model of experimental periodontitis confirmed that GSDMD deficiency alleviates periodontal inflammation and bone loss (13). Moreover, CDK9 inhibition decreases necroptosis and reduces inflammatory bone resorption in mice with bacteria-induced periodontitis (99). Interestingly, experiments in a subcutaneous chamber model showed that the hosts’ ability to clear pathogens improves after inhibition of necroptosis. These findings indicate that PANoptosis plays a pivotal role in the pathogenesis of periodontitis by increasing IL-1β release.

Taken together, these experimental results suggest that PANoptosis may play a pathogenic role in periodontitis. The three kinds of PCD in GFs are responsible for loss of tissue cells and tissue damage. The destruction of gingival tissue further promotes survival of *P. gingivalis*. The principal reason for this is that *P. gingivalis* lacks the ability to synthesize porphyrin and iron, both of which are necessary nutrients for bacterial metabolism. Therefore, the bacteria must obtain heme (a source of iron) from external sources. Gingival exudate flow returns heme, peptides, and amino acids back to the plaque bacteria *via* OMVs; thus damaged tissue aids pathogen proliferation (100). In addition, PANoptosis induced by *P. gingivalis* in THP-1 cells also influences the pathogenesis of periodontitis. As the main type of immune cell, lytic death of macrophages is an important inflammatory mechanism. Damaged host cells release cytoplasmic compounds (e.g., DAMPs, cytokines, and defensins) into the extracellular milieu, which recruit more neutrophils and leukocytes and initiate adaptive immune responses that perpetuate local inflammation and cause alveolar bone destruction. Since stem cells can self‐renew and differentiate into multiple cell types, they have healing capacity. However, PANoptosis caused by *P. gingivalis* is a key factor that reduces the number of stem cells. From another perspective, persistent inflammation is detrimental to regeneration. Release of IL-1β into tissues generates a pro-inflammatory microenvironment, inhibits osteoblastogenesis, and promotes osteoclastogenesis, all of which exacerbate the pathological damage caused by periodontitis. Preventing death of stem cells can restore expression of osteogenic-related proteins, which may have tremendous therapeutic potential.

PANoptosis reflects the constant competition between the host and microbes, which try to exploit various mechanisms and optimize their survival. The outcome is not always beneficial to the host.

### 3.3 Pulp and Periapical Diseases

Pulp and periapical tissues are aseptic. Under the influence of pathological or iatrogenic factors, pulp may be exposed to microbes through an open pulp chamber or openings in the dentinal tubules, leading to pulp infection. When a large bacterial infection exists for a long time, the bacteria pass through the apical foramen, invade the periapical tissue, and cause apical periodontitis (AP). AP is characterized by persistent inflammation and progressive bone destruction. Immune cells and osteoblasts are critical for development of inflammation and repair of bone destruction associated with AP. AP can be divided into primary infections and persistent infections. The dominant bacteria are also different. In primary infections, the most common bacteria are *Fusobacterium nucleatum* (*F. nucleatum*), *Porphyromonas endodontalis*, *Peptostreptococcus micros*, *Campylobacter rectus*, *Prevotella intermedia*, and *Peptostreptococcus anaerobius* (101). With respect to persistent AP, the scientific literature focuses on the role of the *enterococci* (i.e., *Enterococcus faecalis* (*E. faecalis*), is a Gram‐positive optional anaerobic bacterium) (102). Here, we focus on *F. nucleatum* and *E. faecalis* as representative examples.

#### 3.3.1 *F. nucleatum* May Induce PANoptosis in Macrophages

*F. nucleatum* culture supernatants induce apoptosis of human monocyte/macrophage cell lines THP-1 and U937 (65, 74). Y Kawahara (2020) observed mature forms of IL-1β and caspase-1 in THP-1 macrophage-like cells stimulated with *F. nucleatum*. Release of IL-1β from THP-1 macrophage-like cells was inhibited by pretreatment with MCC950(an NLRP3 inhibitor) or z-YVAD-FMK (a caspase-1 inhibitor), suggesting involvement of the NLRP3 inflammasome (75). New evidence shows that *F. nucleatum* induces macrophage necroptosis. In 2021, Liu et al. showed that *F. nucleatum*-OMVs promote the differentiation of pro-inflammatory macrophages and accelerate necroptosis of IECs by activating FADD/RIPK1/caspase-3 signals. The results of co-immunoprecipitation studies revealed the interaction between RIPK1 and RIPK3 in *F. nucleatum* OMVs-treated cells. This was abrogated by anti-TNF-α or Nec-1, indicating that *F. nucleatum*-OMVs upregulate RIPK1 and RIPK3 and promote formation of the necrosome, ultimately causing necroptosis and impairing epithelial barrier function (76).

Taken together, the above studies did not detect simultaneous activation of pyroptosis, apoptosis, and necroptosis; rather, they focused on each mode independently. We can infer that THP-1 cells recognize *F. nucleatum* and engage in a rapid response by producing inflammatory mediators and activating PANoptosis. In the meantime, the host upregulates the anti-apoptotic molecules superoxide dismutase 2 (SOD2) and baculoviral IAP repeat-containing protein 3 (BIRC3) in response to *F. nucleatum*. SOD2 and BIRC3 are anti-apoptotic proteins that act by clearing ROS and inhibiting caspase-3 activity (103). This functions as a protective mechanism that prevents excessive apoptosis during infection.

#### 3.3.2 *E. faecalis* May Induce PANoptosis in Osteoblasts

An *in vitro* study showed that *E. faecalis* induces apoptosis and pyroptosis in human osteoblastic MG63 cells in a MOI-dependent manner. Mechanically, *E. faecalis* increased the Bax/Bcl-2 protein ratio and caspase-3 activity, along with elevation of caspase-1 p20 and the NLRP3 inflammasome. When the NLRP3 inflammasome was downregulated using siRNAs, cells were protected from apoptotic and pyroptic death (77). These results indicate that NLRP3 is involved in apoptosis and pyroptosis of MG63 cells. An *in vivo* study showed that sealing *E. faecalis-LTA* within the pulp of Sprague-Dawley rats resulted in damage to the root apex and periapical bone resorption. Immunohistochemical data revealed upregulated expression of NLRP3, caspase-1, and IL-1β in periapical inflamed tissues (78). Later, other researchers found that *E. faecalis* induces necroptosis of MG63 cells *via* the RIPK3/MLKL signaling pathway. In addition, silencing of MLKL by shRNA or inhibitors reduced cell death rates significantly (79). In conclusion, *E. faecalis* may induce PANoptosis of osteoblasts, which is detrimental to regeneration of periapical bone tissue. However, these two studies used only siRNA or shRNA to downregulate expression of NLRP3 and MLKL, respectively, and the researchers concluded that although partial blocking of pyroptosis or necroptosis pathways reduces the amount of cell death, it cannot completely prevent cells from being killed. Further studies in gene knockout cells (such as caspase-1-/- caspase-8-/- RIPK3-/- cells) are needed to prove whether death of osteoblasts can be prevented completely after blocking PANoptosis.

#### 3.3.3 Significance of PANoptosis in Periapical Periodontitis

PCD is considered to play a role in the pathogenesis of periapical tissue infection. Indeed, expression of mRNA encoding NLRP3, AIM2, caspase-1, and IL-1β is increased in periapical lesions (9). *In vivo* experiments showed that oral infection of mice with *F. nucleatum* results in macrophage recruitment to the dental pulp, as well as bone resorption (104). Microbial pathogens activate inflammasomes and promote production of pro-inflammatory cytokines. Cheng et al. established an experimental model of AP in rats and found that pyroptosis played a role in the formation of periapical lesions (12). Moreover, inhibition of caspase-1 partly decreased bone resorption by suppressing production of IL-1β, MCP-1, IL-6, and IL-8. A recent study revealed that necroptosis may be involved in AP progression (14). Fn-induced AP lesions in Balb/c mice displayed apical bone loss, an increased number of osteoclasts, an enhanced expression of pMLKL, and an increased expression of mRNA encoding inflammatory cytokines. RIPK3 inhibition ameliorated expression of inflammatory cytokines and bone resorption.

Excessive inflammatory response by immune cells is a main cause of bone resorption and delayed healing. Osteoblasts are the initiators of bone remodeling. Owing to the consistent pathological factors (including bacteria) in periodontal tissue, osteoblasts tend to become senescent and, subsequently, dysfunctional. Bacteria-induced PANoptosis helps to inhibit mineralization and alveolar bone formation. Maintaining the activity and function of osteoblasts is important for regeneration of periapical bone. Thus, regulating activation of PANoptosis may be a potential target for preventing disease progression. Unlike osteoblasts, the primary function of macrophages is removal of invading bacteria from cells; thus macrophages promote clearance of pathogens by combined engagement of pyroptosis, apoptosis, and necroptosis. Further studies should examine whether inhibition of PANoptosis affects the clearance of bacteria. Moreover, it is unclear whether upstream signals such as ZBP1 and TAK1 regulate *F. nucleatum*- and *E. faecalis*-induced PANoptosis of macrophages and osteoblasts.

## 4 Summary and Perspectives

### 4.1 PANoptosis Is an Elaborate Host Innate Immune Strategy

PANoptosis has evolved to be a robust fail-safe mechanism activated in response to fungal, viral, and bacterial infections (105). The host can induce any of the three mechanisms of PANoptosis (pyroptosis, apoptosis, and necroptosis) to fulfill its defensive functions, especially when a single arm is inhibited by a pathogen’s immune evasion strategy. Although not all microorganisms trigger PANoptosis, this review summarizes several oral pathogens that may induce PANoptosis (26, 85). Moreover, PANoptosis not only occurs in immune cells, but also in other tissue cells such as fibroblasts, stem cells, and osteoblasts. From the perspective of molecular mechanisms, there is likely a functional redundancy between molecules involved in the complex, which allows for key functions to be carried out even when a specific protein is lost (85). However, the complex interplay between PANoptosome components during specific infections and inflammatory diseases needs further clarification.

### 4.2 PANoptosis Plays a Pluripotent Role in Natural Defense

PANoptosis plays a protective role against infection by fungi and viruses but may have pathogenic effects during a bacterial infection (89, 99). The reason for this paradox may be the different pathogenic mechanisms triggered by fungi, viruses, and bacteria. Fungi are opportunistic pathogens with relatively weak virulence. PANoptosis, a key and conserved system of innate immunity, provides an efficient means of relieving the fungal burden. In terms of viruses, their survival and proliferation depend entirely on the intracellular environment of the host cell. Cell death caused by PANoptosis is detrimental to virus replication (90). Bacteria have strong inflammatory effects due to a wide range of PAMPs and virulence factors.

### 4.3 New Cognition of Pattern Recognition Receptors

NLRs are considered to be intracellular recognition receptors that serve as a first line of host defense. In particular, the NLRP3 inflammasome has been studied extensively over the past two decades. However, there is no consensus regarding whether NLRP3 binds to many stimuli (33). In some cases, ZBP1 is the sensor that identifies microorganisms; it is located upstream of the NLRP3 complex. Furthermore, it is unclear how the PANoptosome is formed. Further studies need to investigate whether other inflammasomes sharing similar domains (e.g., NLRC4 and NLRP6) can be recruited to the PANoptosome (106, 107).

### 4.4 Co-Regulators of the Three Cell Death Pathways May Be Potential Molecular Targets for Therapy

It is revealed that in addition to the direct role of infections, infection-mediated cytokines release could induce PANoptosis (92). When a cytokine storm occurs and causes detrimental inflammation and tissue damage, it is important to note that it may be necessary to target all these cell death pathways simultaneously to control cytokines release. When PANoptosis is activated, blocking just one arm (i.e., blocking just pyroptosis or apoptosis or necroptosis alone) will not prevent cells from dying or releasing inflammatory cytokines. Further investigation of master regulators of PANoptosis may shed some light on modulation of cell death and reduction of cytokine levels. It remains unclear whether it is feasible to control infection and minimize inflammatory pathology *via* PANoptosis. Also, we still do not know if PANoptosis can be induced by additional sensors that are activated in response to other pathogenic challenges. In addition, studies concerning signaling pathways downstream of PANoptosis should be considered. Loss- or gain-of-function of PANoptosis results in dysregulated immune responses. Therefore, the degree to which PANoptosis limits inflammation and controls the bacterial burden is unclear.

Overall, based on emerging efforts that revealing some links between pathogen induced-PANoptosis and oral infectious diseases, PANoptosis highlights the essential role of cell death during microbial infection, providing us with a more comprehensive and profound understanding of pathogenesis of infectious diseases. Meanwhile, improved knowledge of the interplay between the host and pathogens through PANoptosis advancing our understanding toward immunology. Furthermore, the study of PANoptosis is conducive to developing potential molecular therapeutic strategies that target oral infectious diseases.

## Author Contributions

WJ and ZD wrote the original manuscript. XD searched some of the literature. WZ provided the general idea and edited the manuscript. All authors contributed to the article and approved the submitted version.

## Funding

This study was supported by grants from the President Foundation of Nanfang Hospital, Southern Medical University (2019Z019); the Clinical Research Program of Southern Medical University (LC2019ZD023); the Clinical Research Program of Nanfang Hospital, Southern Medical University (2020CR029); and the National Natural Science Foundation of China (82100997).

## Conflict of Interest

The authors declare that the research was conducted in the absence of any commercial or financial relationships that could be construed as a potential conflict of interest.

## Publisher’s Note

All claims expressed in this article are solely those of the authors and do not necessarily represent those of their affiliated organizations, or those of the publisher, the editors and the reviewers. Any product that may be evaluated in this article, or claim that may be made by its manufacturer, is not guaranteed or endorsed by the publisher.
